# Cross-sectional estimates revealed high HIV incidence in Botswana rural communities in the era of successful ART scale-up in 2013-2015

**DOI:** 10.1371/journal.pone.0204840

**Published:** 2018-10-24

**Authors:** Sikhulile Moyo, Simani Gaseitsiwe, Terence Mohammed, Molly Pretorius Holme, Rui Wang, Kenanao Peggy Kotokwe, Corretah Boleo, Lucy Mupfumi, Etienne Kadima Yankinda, Unoda Chakalisa, Erik van Widenfelt, Tendani Gaolathe, Mompati O. Mmalane, Scott Dryden-Peterson, Madisa Mine, Refeletswe Lebelonyane, Kara Bennett, Jean Leidner, Kathleen E. Wirth, Eric Tchetgen Tchetgen, Kathleen Powis, Janet Moore, William A. Clarke, Shahin Lockman, Joseph M. Makhema, Max Essex, Vlad Novitsky

**Affiliations:** 1 Botswana Harvard AIDS Institute Partnership, Gaborone, Botswana; 2 Department of Immunology and Infectious Diseases, Harvard T.H. Chan School of Public Health, Boston, Massachusetts, United States of America; 3 Department of Population Medicine, Harvard Pilgrim Health Care Institute, Boston, Massachusetts, United States of America; 4 Department of Biostatistics, Harvard T.H. Chan School of Public Health, Boston Massachusetts, United States of America; 5 Harvard Medical School, Boston, Massachusetts, United States of America; 6 Division of Infectious Diseases, Brigham and Women's Hospital, Boston, Massachusetts, United States of America; 7 Botswana Ministry of Health and Wellness, Gaborone, Botswana; 8 Bennett Statistical Consulting, Inc., Ballston Lake, New York, United States of America; 9 Goodtables Data Consulting, Norman, OK, United States of America; 10 Department of Epidemiology, Harvard T.H. Chan School of Public Health, Boston, Massachusetts, United States of America; 11 Departments of Internal Medicine and Pediatrics, Massachusetts General Hospital, Boston, Massachusetts, United States of America; 12 U.S. Centers for Disease Control, Atlanta, Georgia, United States of America; 13 Johns Hopkins Hospital, Baltimore, MD, United States of America; Boston University, UNITED STATES

## Abstract

**Background:**

Botswana is close to reaching the UNAIDS “90-90-90” HIV testing, antiretroviral treatment (ART), and viral suppression goals. We sought to determine HIV incidence in this setting with both high HIV prevalence and high ART coverage.

**Methods:**

We used a cross-sectional approach to assessing HIV incidence. A random, population-based sample of adults age 16–64 years was enrolled in 30 rural and peri-urban communities as part of the Botswana Combination Prevention Project (BCPP), from October 2013 –November 2015. Data and samples from the baseline household survey were used to estimate cross-sectional HIV incidence, following an algorithm that combined Limiting-Antigen Avidity Assay (LAg-Avidity EIA), ART status (documented or by testing ARV drugs in plasma) and HIV-1 RNA load. The LAg-Avidity EIA cut-off normalized optical density (ODn) was set at 1.5. The HIV-1 RNA cut-off was set at 400 copies/mL. For estimation purposes, the Mean Duration of Recent Infection was 130 days and the False Recent Rate (FRR) was evaluated at values of either 0 or 0.39%.

**Results:**

Among 12,610 individuals participating in the baseline household survey, HIV status was available for 12,570 participants and 3,596 of them were HIV positive. LAg-Avidity EIA data was generated for 3,581 (99.6%) of HIV-positive participants. Of 326 participants with ODn ≤1.5, 278 individuals were receiving ART verified through documentation and were considered to represent longstanding HIV infections. Among the remaining 48 participants who reported no use of ART, 14 had an HIV-1 RNA load ≤400 copies/mL (including 3 participants with ARVs in plasma) and were excluded, as potential elite/viremic controllers or undisclosed ART. Thus, 34 LAg-Avidity-EIA-recent, ARV-naïve individuals with detectable HIV-1 RNA (>400 copies/mL) were classified as individuals with recent HIV infections. The annualized HIV incidence among 16–64 year old adults was estimated at 1.06% (95% CI 0.68–1.45%) with zero FRR, and at 0.64% (95% CI 0.24–1.04%) using a previously defined FRR of 0.39%. Within a subset of younger individuals 16–49 years old, the annualized HIV incidence was estimated at 1.29% (95% CI 0.82–1.77%) with zero FRR, and at 0.90% (95% CI 0.42–1.38%) with FRR set to 0.39%.

**Conclusions:**

Using a cross-sectional estimate of HIV incidence from 2013–2015, we found that at the time of near achievement of the UNAIDS 90-90-90 targets, ~1% of adults (age 16–64 years) in Botswana’s rural and peri-urban communities became HIV infected annually.

## Introduction

Botswana has been hard hit by the HIV-epidemic, with the third highest HIV prevalence worldwide among adults age 15–49, after Lesotho and Swaziland [1]. Botswana appears to be approaching the UNAIDS “90-90-90” HIV testing, treatment, and viral suppression targets [2]. These high levels of coverage have led to significant reductions in HIV-related mortality [1, 3–5]. In June 2016 Botswana adopted the World Health Organization (WHO) recommendation to provide Universal Test and Treat (UTT) [6]. The success of UTT could be measured by reduction in HIV incidence [7–25]. Monitoring of HIV incidence is a critical tool for assessment and evaluation the impact of HIV prevention and treatment programs.

Prospective longitudinal cohorts remain the gold standard for assessing HIV incidence. However, this approach is time consuming, costly and prone to selection and observational biases [8, 26]. Biomarkers of recent HIV infection that can be detected in cross-sectional samples represent a viable alternative to longitudinal cohort studies. Serological and molecular biomarkers could be combined in multi-assay algorithm (MAA). An optimized MAA with high sensitivity and specificity can discriminate between recent and established HIV infections in cross-sectional sample [27–29][8, 9, 25, 30–36]. Recent advances in design and development of MAA have facilitated estimating of HIV incidence in cross-sectional surveys with improved accuracy [7, 16, 22, 37].

In this study, we estimated HIV incidence using a baseline cross-sectional sample from the Botswana Combination Prevention Project (BCPP; the *Ya Tsie* study) [2, 38, 39]. BCPP is an ongoing pair-matched, cluster-randomized clinical trial in 30 rural and peri-urban communities across Botswana. The primary question of the study is reduction in the cumulative HIV incidence as a result of combination prevention interventions that included enhanced HIV testing and counseling (HTC) campaigns, linkage to care, antiretroviral treatment (ART), strengthened male circumcision (MC) and enhanced prevention of mother-to-child transmission of HIV. Between October 2013 and November 2015, we selected a random sample from 30 communities in three main geographic areas in Botswana: (1) south east, (2) north east, and (3) central eastern region. The communities were purposively selected and proposed based on (1) desired size, and (2) feasibility. Pairs of communities were matched by size, health services, population age structure, and geographic location. In each community, a complete list of all household-like structures (located within the prespecified community boundaries as defined by the 2011 Botswana Census) was obtained and geocoded using satellite imagery (Google Earth, Mountain View, CA, USA). Based on these lists, a simple random sample of approximately 20% of all households was drawn. At each selected household all household members were enumerated, assessed for eligibility, and approached for participation [2]. The vast majority of HIV-positive participants of the baseline household survey, 83%, knew their HIV status [2], 87% of them were receiving ART [2], and 96% of those on ART were virologically suppressed [2].

## Materials and methods

### Study participants

Blood specimens were collected during the BCPP baseline household survey. The HIV-positive status of participants was based on either written documentation provided (e.g., HIV test results, ART prescription) or HIV testing that was performed in the households according to the Botswana national guidelines by using double positive rapid HIV testing. Participants who self-reported not being on ART and classified as recently infected by the MAA were tested for presence of ARV drugs in their plasma. In addition to HTC, the survey staff provided point-of-care CD4 testing, collected blood from people living with HIV for viral load testing and viral genotyping (venous blood was collected by phlebotomy in households), evaluated uptake of HTC, and assessed ART and MC coverage.

Among 12,610 individuals participating in the baseline household survey, HIV status was available for 12,570 participants and 3,596 of them were HIV positive. The study was conducted in accordance with the Declaration of Helsinki. The study received institutional review board approval from the Botswana Health Research Development Committee and the U.S. Centers for Disease Control and Prevention. All participants provided written informed consent. Participants aged 16–18 years provided written assent (with parents or guardians providing written permission). The study is registered at ClinicalTrials.gov (NCT01965470).

### Limiting Antigen Avidity assay and HIV recent infection algorithm

**Fig 1:**
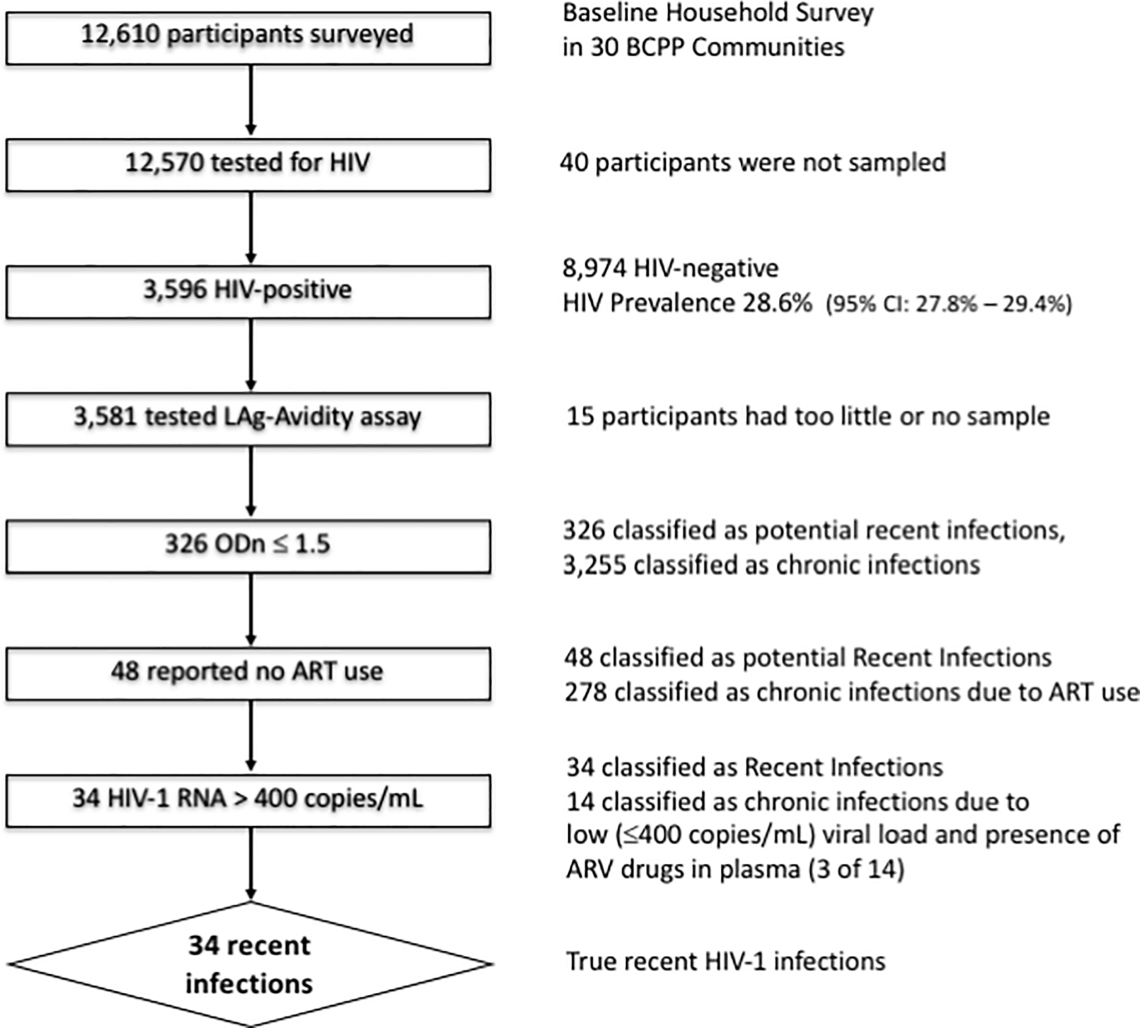
Multiassay algorithm applied for the cross-sectional HIV incidence estimation in Botswana Combination Prevention project.

All plasma specimens from HIV-positive individuals who participated in the survey were tested using the Sedia HIV-1 Limiting Antigen (LAg)-Avidity EIA (Sedia Biosciences Corporation, Portland, OR, USA) according to manufacturer’s instructions [40]. The LAg-Avidity EIA differentiates between ‘recent’ and long-term HIV infection. A normalized optical density (ODn) of <1.5 was considered to represent recent infection [36]. ART status was verified through documentation provided by the participants or testing for presence of ARV drugs in plasma. The MAA [25] included the following steps: (1) plasma specimens from HIV-positive individuals were tested by LAg-Avidity EIA following manufacturer’s recommendations, and ODn was calculated; (2) cases with ODn ≤1.5 were checked for ART status and individuals on ART were excluded from HIV recency candidates; (3) levels of HIV-1 RNA were checked for the remaining candidates and individuals with undetectable viral load (≤400 copies/mL) were excluded from HIV recency candidates; and (4) HIV-positive individuals with ODn ≤1.5 in LAg-Avidity EIA, not taking ARV and having HIV-1 RNA >400 copies/mL were considered recently infected with HIV. Fig 1 shows the MAA applied for the cross-sectional HIV incidence estimation in BCPP. The MAA used the LAg-Avidity EIA in combination with testing for ARV and HIV-1 RNA load. The final sample of recently HIV infected individuals was identified based on HIV-positive status, ODn ≤1.5 in LAg-Avidity EIA, no use of ARVs and detectable viral load (HIV-1 RNA >400 copies/mL) at the time of testing.

### HIV-1 RNA quantification

The HIV-1 RNA load in plasma was quantified by Abbott m2000sp/Abbott m2000rt (Wiesbaden, Germany). HIV-1 RNA >400 copies/mL was considered detectable viral load.

### ARV drug testing

Plasma samples from participants who were classified as recently infected and had undetectable viral load (≤400 copies/mL) were screened for ARV drugs by high-throughput liquid chromatography coupled with Q-Exactive high-resolution mass spectrometry using data-dependent fragmentation and selected reaction monitoring at resolution of 17,500 [41]. To obtain qualitative results, each specimen was compared to positive and negative controls for each drug (abacavir, amprenavir, atazanavir, darunavir, efavirenz, emtricitabine, indinavir, lamivudine, lopinavir, maraviroc, nelfinavir, nevirapine, raltegravir, rilpivirine, ritonavir, saquinavir, stavudine, tenofovir, tipranavir, and zidovudine). The limit of identification ranged from 5 to 10 ng/ml for most drugs and is presented elsewhere [41].

### Statistical analysis and estimation of HIV incidence

The annualized HIV incidence and 95% confidence intervals (CI) were estimated based on cross-sectional incidence assay-based methods that entail biomarkers of HIV disease pro-gression that can distinguish recent from long-term infections [7, 14, 42–44]. Specifically, the annual incidence was estimated as follows:

$$\hat{I}=\frac{n_{R}-{\hat{\beta }}_{T^{n_{+}}}}{n_{s}({\hat{\Omega }}_{T}-{\hat{\beta }}_{T}T)},$$

Where *n_R_*, *n*_+_, and *n_s_* represents the number of individuals who were classified as recent infection, who were HIV-positive (including both recent and non-recent infections), and who were uninfected, in the cross-sectional sample, respectively. ${\hat{\Omega }}_{T}$ is the mean duration of recent infection (MDRI), which is the population average time spent in the ‘recent’ state; and ${\hat{\beta }}_{T}$ is thefalse-recent rate (FRR), representing the proportion of subjects who had been infected for longer than time T (set to be 730 days) but were misclassified as recent infections. MDRI was set to 130 days with a standard error of 5.98 days, corresponding to an ODn threshold of 1.5 [36, 45]. Using HIV-1 RNA load measurement and excluding 14 virologically suppressed individuals from recency candidates justified setting the false-recent rate (FRR) at zero [25, 46]. Missing LAg-Avidity EIA test results were considered missing completely at random. Confidence intervals were estimated taking into account of clustering of communities by applying a design effect to both HIV prevalence and proportion of recent infections among HIV positive individuals, implemented in the R package Inctools v. 1.0.10 [44]. The IncTools implements the HIV incidence calculations from cross-sectional surveys following the guidelines as proposed by the WHO Incidence assays technical working group [23–25, 47].

In addition to estimating HIV incidence among 16–64 years old participants, we assessed HIV incidence in a subset of younger participants in order to compare our results with other studies in Botswana. Specifically, we estimated HIV incidence in a subset of 16–49 years old participants (n = 10,164) including 2,798 HIV-positive and 7,366 HIV-negative individuals.

## Results

A total of 3,596 (29%) individuals from 30 communities in Botswana were HIV positive among 12,570 adults 16–64 years old with definitive HIV status during the baseline household survey of the BCPP from 2013 to 2105 [2]. Table 1 presents basic socio-demographic and clinical characteristics of individuals participating in the baseline household survey. The median (IQR) age was 40 (33–48) years. The majority of participants were females (73%). Among HIV-positive participants, 3,581 (99.6%) were tested by the LAg-Avidity EIA.

**Table 1:** Summary of baseline characteristics of HIV-infected participants enrolled in BCPP in 30 Botswana communities.

Group			Total N (%)		Median CD4+ T cell count ^a^, N (IQR)		Undetectable HIV RNA load (≤400 copies/mL), N (%)	Recent HIV-1 cases, n (%^b^)
All participants			3,596	(100%)	n	408 (259, 596)	2,656 (74%)	34 (0.95%)
Gender								
		Male	962	(27%)	280	337 (200, 525)	665 (25%)	10 (1.0%)
		Female	2,634	(73%)	666	446 (287, 624)	1,991 (75%)	24 (0.9%)
Age group, years								
	16 to 19		41	(1%)	21	524 (392, 708)	16 (39%)	3 (7.3%)
	20 to 29		436	(12%)	220	449 (289, 596)	217 (50%)	19 (4.4%)
	30 to 39		1,185	(33%)	351	427 (258, 627)	845 (72%)	7 (0.6%)
	40 to 49		1,095	(31%)	217	380 (251, 523)	871 (80%)	5 (0.5%)
	50+		839	(23%)	137	384 (247, 554)	707 (84%)	0

Abbreviations: IQR, interquartile range.

^a^ CD4 cell counts among participants not on ART; n indicates the number of participants per group.

^b^ Percent calculated of the total number in the group.

A subset of 326 participants were classified as LAg-Avidity EIA-recent HIV infections with ODn ≤1.5 (Fig 1). The documented ART status was considered as an indicator of long-term HIV infection, and 278 of 326 participants were excluded from recency candidates due to being on ART. A subset of 14 individuals who reported no prior use of ART (including 3 cases with detected ARV drugs in plasma) had undetectable HIV-1 RNA load (≤400 copies/mL), were excluded from the pool of individuals classified as recent HIV-infection [46]. Three of 14 individuals with undetectable viral load and reporting no prior ART use had ARVs in plasma that were the first-line treatment regimens most commonly prescribed in Botswana’s national ART program at the time of sampling: two cases of zidovudin/3TC/efavirenz and one case of zidovudin/3TC/nevirapine. Thus, 34 LAg-Avidity EIA-recent and ARV-naïve participants with detectable HIV-1 RNA load were classified as recent HIV infections (Fig 1). The estimate of annualized HIV incidence is 1.06% (95% CI 0.68–1.45%), assuming an FRR of zero. For a more conservative estimate, we used the adjusted FRR at 0.39% that was determined in our recent study in Botswana [48], and estimated the annualized HIV incidence at 0.64% (95% CI 0.24–1.04%). A higher proportion of recent infections were among young participants, less than 30 years of age (Table 1).

All recently infected individuals (n = 34) were younger than 49 years old. The annualized HIV incidence in the subset of 16–49 years old individuals was estimated at 1.29% (95% CI 0.82–1.77%) with FRR set to zero, and at 0.90% (95% CI 0.42–1.38%) with FRR set to 0.39%. For comparison, two alternative published estimates of HIV incidence in Botswana including UNAIDS [49] are presented in Table 2 along with results of this study.

**Table 2:** Estimates of annualized HIV-1 incidence in Botswana.

Estimate Source	Year	Age group (years)	Point estimate, %	95% CI
UNAIDS modeling [49]	2016	15–49	0.93	0.68–1.18
MAA, LAg-Avidity EIA, FRR = 0, this study	2013–2015	16–64	1.06	0.68–1.45
MAA, LAg-Avidity EIA, FRR = 0.39%, this study	2013–2015	16–64	0.64	0.24–1.04
MAA, LAg-Avidity EIA, FRR = 0, this study	2013–2015	16–49	1.29	0.82–1.77
MAA, LAg-Avidity EIA, FRR = 0.39%, this study	2013–2015	16–49	0.90	0.42–1.38
AIDS Impact Survey IV, BED, FRR = 2.98 [50]	2012	1.5+	1.35	0.43–2.27

Abbreviations. UNAIDS: Joint United Nations Program on HIV/AIDS. MAA: Multi-Assay Algorithm including viral load (400 copies/mL cut-off) and documented HIV status. LAg-Avidity EIA: Limiting Antigen–Avidity EIA. BED–BED Incidence Assay. FRR: False Recent Rate; CI–Confidence Intervals.

## Discussion

HIV incidence in a population-based sample of adults 16–64 years old residing in 30 communities across Botswana was estimated at about 1% from cross-sectional sampling that occurred in 2013–2015. Estimated HIV incidence was slightly higher (0.90–1.29%, depending on the FRR) in a subset of younger 16–49-year-old adults. Results of our study corroborate the recent UNAIDS estimates of HIV incidence in Botswana (0.93%) [49], and suggest a declining trend from previously estimated HIV incidence among 15–49 year old adults in Botswana (3.5% in 2000, 2.4% in 2007 and 1.7% in 2008 [51]). Our results support the observation that new HIV infections across sub-Saharan Africa continue to decline, although HIV incidence in Botswana remains unacceptably high [52].

The strength of the current estimate of HIV incidence includes population-based random sampling from 30 rural and peri-urban communities across the country, and application of MAA that includes LAg-Avidity EIA, ART status, and measurements of HIV-1 RNA in all HIV-positive participants. Limitations of our study include MAA that is reliant upon the ART status and the uncertainty arising from estimates of corresponding FRR and MDRI. In our previous studies, we have also found similar estimates of MDRI using an MAA with ART status and viral load [53]. Individuals on ART were excluded from recency candidates, because being on ART was interpreted as an indicator of longstanding HIV infection. This approach worked well in the era of CD4-driven initiation of ARV therapy, and was in line with the Botswana HIV treatment guidelines at the time of sampling, 2013 to 2015. In June 2016 Botswana introduced a new national policy “Treatment for All”, that is, initiating ART as soon as possible regardless of CD4+ T-cell counts [6]. The ongoing scaling up of this national policy means that ART status cannot be used as exclusion criteria for estimation of HIV recency in future studies. Novel cross-sectional assays and MAA independent of ART status are needed to address this issue.

Self-reported ART status could be considered one of the study limitations. Although ART status was verified through documentation among those who self-reported to be on ART, there is an uncertainty due to possible undisclosed ART use among those who self-reported not to be on ART. To address this limitation we performed ARV drug testing in plasma among those who had undetectable levels of HIV-1 RNA and reported no ART use. In fact, we found triple ARV drugs in 3 out of 14 cases. Using viral load threshold could minimize uncertainty of self-reported status. In fact, 3 cases with ARV drugs in plasma were excluded based on low levels of HIV-1 RNA.

While we used a Botswana-specific FRR from our previous study [48], it was estimated in a cohort sampled approximately a decade before sampling in the current study. We speculate that FRR in Botswana could be decreasing over time and could be lower than the 0.39% used for conservative estimates of HIV incidence in this study. The extent to which regional FRRs are changing over time remains unknown. The scale up of national ART programs could affect FRR estimates. Since ART guidelines have been changing, a greater proportion of individuals are initiating treatment sooner, more frequently soon after HIV diagnosis. This means the increasing ART coverage could reduce FRR. In this manuscript, the MAA algorithm included ARV drug testing leading to reduction of FRR to zero. However, uncertainly remains as to whether FRR can be eliminated [47]. The upper range of FRR then is FRR without drug tracing, as we determined in our previous study in Botswana [48, 54].

The relatively high estimated HIV incidence that we found (~1%) despite high levels of HIV testing, treatment, and viral suppression may reflect several factors. First, the impact of widespread ART on HIV incidence may take several years to be realized. In addition, the ~30% of HIV-infected individuals with detectable viremia [2] could yield such high HIV incidence in the setting of very high HIV prevalence (and these individuals could have different HIV risk behavior, compared with those with viral suppression on ART). Our findings highlight the importance of targeted interventions to reach individuals who have not yet sought HIV testing or treatment services.

## Conclusion

In summary, using cross-sectional sampling and MAA based on LAg-Avidity EIA, ART status (either documented or by testing ARV drugs in plasma) and HIV-1 RNA measurements, we estimated the HIV incidence in 30 rural and peri-urban Botswana communities in 2013–2015 at about 1%. A higher proportion of recent infections were among participants less than 30 years of age. A reduction from this relatively high estimated HIV incidence may take several years to be realized despite the impact of widespread ART and other on-going interventions. Targeted interventions are required to reach individuals who have not yet sought HIV testing or treatment services.
